# The Communication of Multiple Sclerosis Diagnosis: The Patients' Perspective

**DOI:** 10.1155/2015/353828

**Published:** 2015-12-15

**Authors:** Maria Josè Messina, Gloria Dalla Costa, Mariaemma Rodegher, Lucia Moiola, Bruno Colombo, Giancarlo Comi, Vittorio Martinelli

**Affiliations:** Institute of Experimental Neurology, Division of Neuroscience, Scientific Institute and University San Raffaele Hospital, Via Olgettina 48, 20132 Milan, Italy

## Abstract

*Background*. Multiple sclerosis (MS) is the leading cause of nontraumatic neurological disability in young adults in Europe and in the United States. The uncertainty regarding its evolution makes the diagnosis disclosure a difficult process.* Objective*. The aim of the study was to provide patients' global perspective towards MS diagnosis communication.* Methods*. 150 consecutive patients, recently diagnosed with CIS or MS, were asked to complete a 17-item questionnaire assessing factors influencing their satisfaction with the information provided.* Results*. Eighty-six patients fulfilled diagnostic criteria for MS and 64 for CIS. Diagnosis disclosure took place in a private setting and required in most cases (87.3%) less than 30 minutes. Most patients reported being moderately or highly satisfied with the information provided (75%). The degree of satisfaction seems significantly related to patients' younger age, a longer time dedicated to disclose the diagnosis, a CIS diagnosis, and, above all, tailored information and an adequate emotional support.* Conclusion*. Most patients reported a good degree of satisfaction about the communication of MS or CIS diagnosis. A fruitful relationship between patient and neurologist is essential to obtain a better acceptance of the disease, patients' compliance with chronic treatments and to improve patients' quality of life.

## 1. Introduction

Multiple sclerosis (MS) is a chronic inflammatory disease of the central nervous system (CNS) and represents the leading cause of nontraumatic neurological disability in young adults in the United States and Europe. The bad common perception of MS, the uncertainty regarding the evolution of the disease, and the response to treatments make the disclosure of the diagnosis a difficult and complex process. In fact, to receive “bad news,” related to an unpredictable condition, can have a big impact on the psychological well-being [1]. Since the first symptoms suggestive of MS, the disclosure of this new physical condition induces patients to a new different self-knowledge process and feelings of anxiety and depressive symptoms are frequent, especially in the first period [2]. An effective diagnosis communication is critical as it may improve acceptance of the disease, patients' compliance with chronic treatments, and, ultimately, patients' quality of life. This is even more important considering that the revised MS diagnostic criteria allow an earlier MS diagnosis [3] and disease modifying treatments (DMTs) are now available just after the clinical onset of the disease [4]; thus more often patients start a DMT before developing a trusting and close relationship with the neurologist [5].

Despite the importance of an effective diagnosis communication, few studies on the current practice about communicating MS diagnosis have been published so far. We thus conducted a study to assess patients' opinions and perspectives regarding the manner in which physicians deliver MS diagnosis and their feedback and suggestions to improve the process.

## 2. Materials and Methods

This was a single-center, observational study conducted between July 2012 and July 2014. We included 150 consecutive inpatients or outpatients who received a diagnosis of clinically isolated syndrome (CIS) or MS at our or other Italian neurological departments. Eligible patients were approached by two of the authors (Vittorio Martinelli or Maria Josè Messina) who outlined the objectives and nature of the study and obtained written informed consent from willing participants. The local ethical committee approved this study as part of a large protocol on patients after the first clinical attack.

An Italian survey was developed for this study in order to assess factors affecting patients' satisfaction with the diagnosis communication. A preliminary version was assembled using the PROFILE project survey [6], which was designed to assess the factors influencing the diagnosis communication process from the Italian neurologist's perspective.

We evaluated patients' perspective using the same questionnaire referred to physicians, but with questions regarding patients' experience, to detect the different views of the two leading actors during the diagnosis communication. Two neurologists, one methodology expert, and an ad hoc focus group of 10 patients reviewed the preliminary version of the questionnaire until it had sufficient face validity to measure the concepts of interest. The final survey was a 17-item questionnaire (see Supplementary Material available online at http://dx.doi.org/10.1155/2015/353828) collecting information on patients' demographics, timing, setting, and terminology used during the diagnosis communication. Patients were also asked about the satisfaction with the information provided, their reactions, emotional profile, and their view regarding communication issues.

Satisfaction with the information provided was recorded using a three-level scale; low, medium, or high. Univariate ordinal logistic regression was fitted between satisfaction and each of the explanatory variables to assess the crude effect of each on satisfaction. Odds ratios were estimated with the lowest category of each explanatory variable used as the reference value. A likelihood ratio (LR) test was performed to assess the overall significance of each explanatory variable. All of the explanatory variables of interest were then included in a multivariable ordinal logistic regression to estimate the adjusted odds ratios. Parameters of the ordinal logistic regression models were estimated with ridge penalization. In order to choose the optimal ridge penalization value, candidate values in the range of 0-1 were assessed based on the resulting misclassification error. For the full model the optimal penalty used was 0.01. All statistical analyses were performed using the computing environment R (R Development Core Team, 2005).

## 3. Results

A total of 150 questionnaires were available for analysis. The demographic characteristics of the participants are listed in Table 1. The median age of patients was 31 years (IQ range 24–38 years). The median time since diagnosis communication was 0 days (IQ range 0–40 days). Most patients received the diagnosis in Northern Italy (81%), and 29 (19%) received the diagnosis in Southern or Central Italy. Eighty-six (57%) patients were affected by MS according to 2010 McDonald criteria and 64 (43%) patients had a diagnosis of CIS at time of study entry. Most patients had low disability, as just 17 (11.3%) patients had an EDSS equal to or higher than 2.0 at the time of the questionnaire. In the presence of a first neurological symptom suggestive of MS, regardless of the geographical area, most patients (76%) had been hospitalized, while just 36 (24%) patients had been investigated through one or more outpatient sessions. Diagnosis disclosure took place in a private setting and required in most cases (87.3%) less than 30 minutes (it took more than 30 minutes for 14 (9.3%) patients and more than 1 hour for only 3 (2%) patients). Patients' family members were often present (104 patients, 69.3%) during the communication of the diagnosis (Table 2).

### 3.1. Factors Affecting Patients' Satisfaction during Diagnosis Disclosure

Around half of the patients reported being moderately satisfied with the information provided at the diagnosis communication (92 patients, 61.3%) and 24 (16%) patients were highly satisfied, while 34 (23.3%) patients were not at all satisfied. The required information referred to MS diagnosis, prognosis, and available treatments.

The univariate analysis showed that most patients highly satisfied were younger, received the diagnosis in Northern Italy, and completed the questionnaire after a shorter period of time from the diagnosis (*p* < 0.01). Patients with a MS diagnosis reported significant lower satisfaction with the information provided than patients with CIS (*p* < 0.01). The setting did not seem to be as important as the spent time, as patients whose disclosure of the diagnosis took at least 30 minutes were the most satisfied. Finally, an adequate emotional support and a communication tailored to the patient's profile resulted in a higher satisfaction (Table 3).

The multivariate ordinal logistic regression model confirmed these results, as most patients highly satisfied with the information provided were younger (*p* = 0.04), received a diagnosis of CIS (*p* < 0.001), experienced an adequate emotional support (*p* < 0.001), and reported a communication tailored to the patient's profile (*p* = 0.058) (Table 4).

### 3.2. Patients' Feedback and Suggestions to Improve the Diagnosis Communication Process

Concerning timing of diagnosis communication, the vast majority of patients (90%) preferred to receive the communication during the diagnostic workup, whereas only 10% chose to postpone the communication after the second neurological episode. In the diagnosis communication, the presence of an expert psychologist in a MS center is perceived as relevant in changing the management of emotionally difficult situations such as the moment of communicating the diagnosis. At the same time, 67.3% of patients found it to be useful to take part in public meeting with other patients sharing their experiences and perspectives. Furthermore, 51.3% of patients believed that an additional information aid could be useful to effectively contribute to improving patients' knowledge about the disease and the available treatments.

## 4. Discussion

The reaction to the diagnosis of a chronic, disabling disease with an unpredictable course such as MS can be very destructive [6–8]. Given this, many neurologists still hesitate to pursue a MS diagnosis since the first symptoms or often use nonspecific terms such as “inflammatory disease” or “multifocal demyelinating disease of the CNS,” as in our study that emerged. Though, a prompt and effective communication of the diagnosis seems to be critical for improving patients' acceptance of the disease and compliance [9–12]. In our study, the vast majority of patients (90%) preferred to receive the communication during the diagnostic workup, whereas only 10% would have chosen to postpone the communication after the second neurological episode. These results confirm the findings of previous studies. In a Greek survey [13, 14] assessing MS patients' opinions about their experience in receiving the diagnosis, 91% of patients referred to the fact that they would prefer to know immediately the diagnosis, but only 44% actually experienced this condition. According to 76.8% of patients, a delay in the disclosure of the diagnosis would decrease their trust in their neurologist. In another study, Brown et al. [15] found that patients who were not adequately informed and could not actively participate in the management of the disease presented an increased risk of depression.

In our study, around 75% of the patients reported being moderately or highly satisfied with the information provided and with the whole communication process they had experienced. The degree of satisfaction was significantly related to a longer time dedicated to the process, except for patients who required more than 60 minutes to be informed, for whom a high level of anxiety, denial towards the disease, or other rare psychological conditions may be supposed. In our study, the setting (place where the diagnosis was disclosed and presence or not of patients' relatives) was not as important as the time devoted to the diagnosis communication for patients' satisfaction. Though, the most important factors influencing patients' satisfaction with the diagnosis were tailored information provision and, above all, an adequate emotional support. These data pieces confirm previously published findings on the importance of emotional support in the disclosure of a MS diagnosis [16]. Indeed, there is a long-standing tension in the physician's role since the twentieth century, with doctors striving for detachment to reliably care for all patients regardless of their personal feelings. Though, clinical empathy has been proven to be a powerful tool that health professionals can use to deliver care that is adapted to patients' emotional, cognitive, and biological needs. Clinical empathy involves the ability to understand the patient's inner experiences and perspective and a capability to communicate this understanding. This ultimately enables strengthening the therapeutic relationship and the patient's trust in the neurologist.

Our results confirm that a dedicated individual approach could help to ensure that patients assimilated and did not misunderstand the information provided, as previously found by Heesen et al. [17]. Nevertheless, patients' emotional state at the moment of the diagnosis makes it harder to understand the information provided, and questions or doubts could arise after the initial communication of the diagnosis. In fact about 50% of our patients reported an information aid could be useful to effectively improve their knowledge on the disease and the available treatments even further. It is well known that many patients, after the communication of the diagnosis, often seek more information on the Internet, so the quality of their knowledge is often misleading or inadequate. Therefore an information aid specifically prepared for newly diagnosed patients could provide the patient with good-quality medical information accessible anytime and anywhere. In a recent Italian multicenter study [18, 19], the effectiveness of an information aid for newly diagnosed MS patients was assessed. Patients who received the material during the trial showed a higher satisfaction with care, compared to patients without information aid. The content was considered safe and anxiety did not increase in the intervention group.

Finally, in our study most patients (67%) suggested public meetings with other patients, sharing directly their experiences and perspectives, could be useful to improve their coping strategies after the communication of the diagnosis. In fact, other patients who have experienced the same situation could have a better understanding of the questions and doubts patients face after the initial diagnosis and could help solve them and reach a better understanding of the medical information provided.

The relatively small sample size and the prevalent involvement of patients from the Northern Italian MS centers may be some limitations of our study, as whether the sample is really representative of Italian MS patients could be questioned. Also, the type of symptoms occurred and the presence of anxiety and depression at baseline was not assessed. These may have an influence on our results, especially considering how close to the onset of symptoms our survey had been administered, and it may prevent drawing definitive conclusions from our study.

Anyway, our study should be considered a pilot experience aimed, first of all, at sensitizing people to this issue and analyzing both the awareness of one's limitations and need for improvement. A thorough analysis of both patient's and neurologist' perspectives on this issue may represent the best option to plan further improvements.

An important extension of the current work would be to prospectively evaluate evolving ways and strategies of disclosing the diagnosis of MS over time (i.e., the impact of new health related technologies) and providing information about the disease that meet patients preferences and expectations.

Such efforts are important, as providing additional individualized news may effectively improve acceptance of MS diagnosis, which may influence not only the adherence to a chronic DMT but also the patients' quality of life.

## Supplementary Material

Supplementary Material. Italian and English version of the Questionnaire used in this study. The questionnaire, a 17-item questionnaire derived from the PROFILE project questionnaire [6], is aimed at collecting information on timing, setting, and terminology used during the diagnosis communication and patients' satisfaction about the disclosure of the diagnosis they experienced.

## Figures and Tables

**Table 1:** Patients' sociodemographic and disease-related characteristics.

	Patients (*n* = 150)
Age at onset, median (IQ range)	30 (24–38)
Gender	
Females, number (%)	101 (67.3)
Males, number (%)	49 (32.7)
Actual disease, ^a^number (%)	
Clinically isolated syndrome	64 (42.7)
Multiple sclerosis	86 (57.3)
Other	0 (0.0)
MS centre location, number (%)	
North of Italy	121 (80.7)
Centre of Italy	7 (4.7)
South of Italy	22 (14.7)
EDSS at the time of diagnosis, median (IQ range)	1.5 (1.0–2.0)
Time from the diagnosis, median (IQ range)	0 (0–40)

^a^Defined by the 2011 diagnostic criteria.

**Table 2:** Descriptive statistics.

Variable	All patients	Patient's satisfaction (%)		
		Low	Medium	High
Age at onset				
15–25	39	17.9	56.4	25.6
25–35	61	21.3	63.9	14.8
35–45	41	31.7	56.1	12.2
45–55	9	11.1	88.9	0.0
Gender				
Male	49	26.5	61.2	12.2
Female	101	20.8	61.4	17.8
Actual disease				
Clinically isolated syndrome	64	14.1	64.0	21.9
Multiple sclerosis	86	29.1	59.3	11.6
Time from the diagnosis				
Less than 30 days	103	16.5	64.1	19.4
More than 30 days	47	36.2	55.3	8.5
MS centre location				
North of Italy	121	18.2	65.3	16.5
Centre of Italy	7	71.4	28.6	0.0
South of Italy	22	31.8	50.0	18.2
EDSS at the time of diagnosis				
0	16	18.8	62.5	18.8
1-2	117	24.8	58.1	17.1
>2	17	11.8	82.4	5.9
Type of patient care				
Inpatient care	114	21.1	62.3	16.7
Outpatient care	19	26.3	52.6	21.1
Day hospital	17	29.4	64.7	5.9
Where did you receive the diagnosis?				
In my hospital room	46	19.6	63.0	17.4
In a private office	91	24.2	58.2	17.6
In a public space	6	0.0	100.0	0.0
Other (by phone or discharge letter)	7	42.9	57.1	0.0
Were other people present at the time of the diagnosis?				
No	31	22.6	67.7	9.7
My family members	105	21.9	59.0	19.0
Other people	14	28.6	64.3	7.1
How long did the communication of the diagnosis take?				
Less than 15 minutes	67	35.8	55.2	9.0
15–30 minutes	65	15.4	66.2	18.5
30–60 minutes	15	0.0	60.0	40.0
More than 60 minutes	3	0.0	100	0.0
Did the neurologist provide you with the adequate emotional support?				
No	31	64.5	35.5	0.0
A little bit	71	16.9	71.8	11.3
Yes	48	4.2	62.5	33.3
Did the neurologist tailor the communication to your profile?				
No	38	50.0	44.7	5.3
A little bit	82	14.6	70.7	14.6
Yes	30	10.0	56.7	33.3

**Table 3:** Univariate analysis of factors affecting patients' satisfaction with the disclosure of the diagnosis.

Variable	OR	Lower	Upper	*p* value	LR
		95% CI	95% CI		*p* value
Age at onset					
15–25	Ref.				
25–35	0.35	0.13	0.95	0.04	0.08
35–45	0.25	0.08	0.76	0.01	
45–55	0.28	0.05	1.66	0.16	
Gender					
Male	Ref.				
Female	1.61	0.68	3.81	0.28	0.24
Actual disease					
Clinically isolated syndrome	Ref.				
Multiple sclerosis	0.57	0.24	1.36	0.21	0.24
Time from the diagnosis					
Less than 30 days	Ref.				
More than 30 days	1.06	0.35	3.20	0.92	0.89
MS centre location					
North of Italy	Ref.				
Centre of Italy	0.14	0.01	1.59	0.11	0.17
South of Italy	1.26	0.36	4.48	0.72	
EDSS at the time of diagnosis					
0	Ref.				
1-2	1.10	0.30	3.92	0.88	0.93
>2	0.87	0.17	4.49	0.87	
Type of patient care					
Inpatient care	Ref.				
Outpatient care	1.70	0.50	5.78	0.39	0.38
Day hospital	0.54	0.14	2.07	0.37	
Where did you receive the diagnosis?					
In my hospital room	Ref.				
In a private office	1.06	0.40	2.85	0.90	0.55
In a public space	0.35	0.05	2.60	0.31	
Other (by phone or discharge letter)	0.33	0.05	2.38	0.27	
Were other people present at the time of the diagnosis?					
No	Ref.				
My family members	0.64	0.23	1.77	0.39	0.50
Other people	0.37	0.07	1.83	0.22	
How long did the communication of the diagnosis take?					
Less than 15 minutes	Ref.				
15–30 minutes	2.19	0.91	5.27	0.08	0.15
30–60 minutes	4.67	1.00	21.87	0.05	
More than 60 minutes	3.14	0.19	51.28	0.42	
Did the neurologist provide you with the adequate emotional support?					
No	Ref.				
A little bit	4.96	1.54	15.96	<0.01	<0.001
Yes	17.72	4.53	69.38	<0.0001	
Did the neurologist tailor the communication to your profile?					
No	Ref.				
A little bit	2.68	0.96	7.49	0.06	0.08
Yes	4.36	1.15	16.45	0.03	

**Table 4:** Multivariate analysis of factors affecting patients' satisfaction with the disclosure of the diagnosis.

Variable	OR	Lower	Upper	*p* value	LR
		95% CI	95% CI		*p* value
Age at onset					
15–25	Ref.				
25–35	0.65	0.30	1.43	0.28	0.26
35–45	0.42	0.18	1.00	0.05	
45–55	0.61	0.15	2.48	0.48	
Gender					
Male	Ref.				
Female	1.43	0.73	2.81	0.30	0.30
Actual disease					
Clinically isolated syndrome	Ref.				
Multiple sclerosis	0.44	0.22	0.85	0.01	0.01
Time from the diagnosis					
Less than 30 days	Ref.				
More than 30 days	0.36	0.18	0.74	<0.01	<0.01
MS centre location					
North of Italy	Ref.				
Centre of Italy	0.10	0.02	0.53	<0.01	<0.01
South of Italy	0.67	0.27	1.66	0.39	
EDSS at the time of diagnosis					
0	Ref.				
1-2	0.79	0.29	2.13	0.64	0.89
>2	0.86	0.23	3.17	0.82	
Type of patient care					
Inpatient care	Ref.				
Outpatient care	0.97	0.37	2.52	0.95	0.51
Day hospital	0.56	0.20	1.52	0.26	
Where did you receive the diagnosis?					
In my hospital room	Ref.				
In a private office	1.17	0.58	2.36	0.67	0.46
In a public space	1.26	0.25	6.34	0.78	
Other (by phone or discharge letter)	0.34	0.08	1.51	0.16	
Were other people present at the time of the diagnosis?					
No	Ref.				
My family members	1.35	0.62	2.95	0.45	0.50
Other people	0.76	0.23	2.56	0.66	
How long did the communication of the diagnosis take?					
Less than 15 minutes	Ref.				
15–30 minutes	2.71	1.33	5.51	<0.01	<0.0001
30–60 minutes	8.63	2.74	27.23	<0.01	
More than 60 minutes	1.94	0.20	18.73	0.57	
Did the neurologist provide you with the adequate emotional support?					
No	Ref.				
A little bit	7.78	3.18	19.04	<0.0001	<0.0001
Yes	31.29	10.74	91.19	<0.0001	
Did the neurologist tailor the communication to your profile?					
No	Ref.				
A little bit	4.53	2.05	10.01	<0.001	<0.0001
Yes	10.44	3.76	29.0	<0.0001	
