# Supplementary material for: The Communication of Multiple Sclerosis Diagnosis: The Patients' Perspective
Source: Mult Scler Int. 2015 Dec 15;2015:353828. doi: 10.1155/2015/353828 (PMC4693007; doi:10.1155/2015/353828)
Supplement: Supplementary file 1 — Supplementary Material. Italian and English version of the Questionnaire used in this study. The questionnaire, a 17-item questionnaire derived from the PROFILE project questionnaire [6], is aimed at collecting information on timing, setting, and terminology used during the diagnosis communication and patients' satisfaction about the disclosure of the diagnosis they experienced. [file 353828.f1.pdf]

Supplementary file

Italian version

QUESTIONARIO "COMUNICAZIONE DELLA DIAGNOSI"

Nome e cognome____

Data di nascita ____

"Comunicare la diagnosi" e' uno degli atti medici più delicati nel rapporto medico-paziente.

Questo questionario è atto a valutare quanto lei sia rimasto soddisfatto da questo momento e, qualora presenti, gli aspetti negativi.

1) **A seguito dell'esordio dei sintomi, il suo neurologo curante ha deciso di disporre per:**
   a. Ospedalizzazione
   b. Ricovero in regime di Day-Hospital
   c. Follow-up ambulatoriale

2) **Nella "comunicazione della diagnosi", quale termine è stato utilizzato:**
   a. Sindrome Clinicamente Isolata (CIS)
   b. Possibile Sclerosi Multipla
   c. Malattia infiammatoria/demielinizzante del Sistema Nervoso Centrale
   d. Altro

3) **Quando pensa sia meglio affrontare la comunicazione della diagnosi da parte del suo neurologo?**
   a. Sin dal sospetto iniziale, cioè dalla prima visita che ho fatto con lui
   b. Già durante il ricovero o il Day Hospital, man mano che si procede con gli esami
   c. È meglio rimandare la comunicazione della diagnosi ad una fase successiva al ricovero o ciclo di Day Hospital

4) **Dopo la comunicazione della diagnosi, preferirebbe:**
   a. Essere subito informato circa l' evoluzione futura dei miei disturbi/malattia
   b. Aspettare ulteriori conferme cliniche sulla diagnosi, prima di pensare alle possibili evoluzioni.
   c. Essere supportato dal punto di vista psicologico.

5) **Ritiene che durante la comunicazione della diagnosi il suo neurologo abbia man mano considerato la sua:**
   a. Emotività poco abbastanza molto
   b. Età poco abbastanza molto
   c. Situazione socio-economico poco abbastanza molto
   d. Situazione psicologica poco abbastanza molto

6) **Cosa ritiene abbia interferito maggiormente durante i momenti in cui ti è stata comunicata la diagnosi?**
   a. Il fattore tempo poco abbastanza molto
   b. Inadeguatezza degli spazi poco abbastanza molto
   c. Difficoltà nello stabilire una relazione tra paziente e medico
      poco abbastanza molto

7) **Come e dove è avvenuta la "comunicazione della diagnosi":**
   a. Nella mia camera di ospedale
   b. In un incontro riservato nello studio medico
   c. In più di un incontro, nello studio medico
   d. In uno spazio comune nel reparto
   e. Telefonicamente
   f. Tramite lettera di dimissione

8) **Quanto tempo è stato dedicato complessivamente alla "comunicazione della diagnosi"?**
   a. Meno di 15 minuti
   b. Tra 15 e 30 minuti
   c. Tra 15 e 60 minuti
   d. Più di un'ora

9) **Chi era presente con lei alla "comunicazione della diagnosi"?**
   a. Nessuno, ero solo/a
   b. Vi era un mio parente (genitore fratello-sorella)
   c. Vi erano 2 o più parenti
   d. Vi erano uno o più altre persone (non parenti)

10) **In genere che cosa ritiene sia preferibile durante il primo incontro in cui le è stata comunicata la diagnosi?**
   a. Avere informazioni sommarie senza entrare troppo nel dettaglio
   b. Essere rassicurati
   c. Massima trasparenza ed essere informato circa le implicazioni future
   d. Avere oltre alla diagnosi una adeguata informazione sulle terapie

11) **Ritiene sia utile la presenza di uno psicologo durante il colloquio di "comunicazione della diagnosi"?**
   a. No, preferisco affrontare da solo il momento della comunicazione della diagnosi.
   b. Sì, ritengo possa essere utile la presenza di uno psicologo per aiutare il neurologo nel comprendere e gestire la modalità di comunicazione con il paziente
   c. Sì, ritengo possa essere utile la presenza di uno psicologo per aiutarmi a gestire i problemi emozionali conseguenti alla comunicazione della diagnosi

12) **Durante la "comunicazione della diagnosi", avrebbe preferito fosse stato utilizzato materiale informativo di supporto?**
   a. No, preferisco ricevere le informazioni necessarie direttamente dal medico
   b. Sì, avrebbe potuto essermi utile per integrare le informazioni ricevute ed avere una migliore comprensione della malattia
   c. No, ho cercato altre informazioni su Internet-altro

13) **Secondo lei, qual è la modalità di comportamento migliore nel rapporto medico-paziente?**
   a. È il medico che deve decidere cosa sia meglio fare in merito sia agli esami sia alla terapia
   b. Il medico deve spiegare il proprio orientamento, ma decidere cosa fare solo dopo aver sentito il parere del paziente, in modo che la scelta sia condivisa
   c. Il medico deve sempre spiegare il proprio orientamento, ma lasciare la decisione finale al paziente senza influenzarlo
   d. Il medico deve spiegare in modo obiettivo le possibili scelte, senza esprimere il proprio parere, in modo da lasciare completamente libero il paziente nella scelta finale

14) **Dopo la comunicazione della diagnosi hai avuto la sensazione di:**
   a. Non aver ricevuto un sufficiente supporto emotivo
   b. Che il medico sia stato abbastanza disponibile nel comprendere e rispondere alle mie aspettative
   c. Che il medico abbia gestito in maniera esaustiva le mie necessità

15) **A suo parere, ritiene che vi sia stata una "comunicazione personalizzata" da parte della equipe che l'ha seguita, durante il processo diagnostico-terapeutico?**
   a) No
   b) Sì, un po'
   c) Sì, discretamente
   d) Sì, molto

16) **Dopo la "comunicazione della diagnosi" ha avuto la consapevolezza di essere stato esaurientemente informato sulla sua malattia?**
   a. No
   b. Sì, un po'
   c. Sì, discretamente
   d. Sì, molto

17) **Parteciperebbe ad incontri di approfondimento sulla sua diagnosi/malattia con altri malati che hanno avuto la sua stessa esperienza?**
   a. Sì, solo con altre persone che hanno avuto il mio stesso problema per eventuali confronti
   b. Sì, ma con la presenza del neurologo
   c. Sì, ma con la presenza dello psicologo
   d. No

English version

“DIAGNOSIS COMMUNICATION” SURVEY

Name and Surname____

Date of Birth ____

The communication of the diagnosis is considered a critical moment of the physician-patient relationship.

This survey is designed to assess the satisfaction and the eventual issues about the diagnostic process.

1) After the onset of symptoms, your neurologist recommended for:

a. Hospitalization

b. Day-Hospital

c. Outpatient follow-up

2) During the communication of the diagnosis your neurologist used the term:

a. Clinically Isolated Syndrome (CIS)

b. Possible Multiple Sclerosis

c. Inflammatory/demyelinating CNS

d. Other

3) When do you think it’s better to receive the communication of the diagnosis?

a. Since the first visit, if clinical condition was suggestive

b. During hospitalization or day hospital, after the diagnostic exams

c. It’s better to put off the communication of the diagnosis till after the end of hospital/Day Hospital stay

4) After the disclosure of the diagnosis, you’d prefer:

a. To be immediately informed about the possible future evolution of my disease

b. To have further clinical and instrumental information confirming the diagnosis before being informed about the possible future evolution of the disease

c. To receive psychological support

5) Do you think that, during the communication of the diagnosis, your neurologist considered:

a. Emotional aspects	little	enough	much
b. Age	little	enough	much
c. Socio-economic state	little	enough	much
d. Psychological condition	little	enough	much

6) What has influenced the most the communication of the diagnosis?

a. time	little	enough	much
b. Inadequate setting	little	enough	much
c. Difficult relationship with the doctor	little	enough	much

7) Where the communication of the diagnosis took place?

a. Room of the hospital

b. Private setting, during a single visit

c. Private setting, during the course of a series of visits

d. Department open-space

e. By phone

f. By discharge letter

8) How much time has the neurologist spent to communicate the diagnosis?

a. Less than 15 minutes

b. Between 15 and 30 minutes

c. Between 15 and 60 minutes

d. More than one hour

9) Was any other person present when you received the diagnosis?

a. None

b. A relative (parent-brother-sister)

c. Two or more relatives

d. one or more other persons (not relatives)

10) What would you prefer to know at the beginning of the disclosure of the diagnosis process?

a. To receive general information with no specific details

b. Reassurance

c. To be informed about possible future implications

d. To receive further information about the diagnosis and possible therapies

11) Do you think the presence of a psychologist during the communication of the diagnosis would be useful?

a. No, I'd prefer to be alone during the diagnosis communication

b. Yes, I think a psychologist could help the neurologist in the disclosure process

c. Yes, I think a psychologist could help me to manage the emotional problems related to the communication of the diagnosis

12) Do you think it could be useful to receive an information aid (leaflet, book, etc) after the disclosure of the diagnosis?

a. No, I prefer to receive information from the neurologist

b. Yes, it could be useful

13) In your opinion, how should the doctor-patient relationship be modelled?

a. The doctor has to choose diagnostic exams to be perfomed and therapeutic strategies

b. The physician should explain his own opinions and come to a decision together with the patient

c. The physician should explain his own opinions, but the patient has the final decision

d. The physician should explain objectively all the diagnostic/therapeutic possibilities and the patient should come to a decision without any conditioning

14) After the disclosure of the diagnosis, you feel like:

a. You have not received sufficient emotional support

b. The doctor understood your emotional state and fulfilled your expectations

c. The doctor met all my needs

15) In your opinion, you believe to have received a "personalized communication"?

a) No

b) Yes, a little

c) Yes, fairly

d) Yes, very much

16) Do you think you have been informed adequately about the disease during the communication of the diagnosis"?

a. No

b. Yes, a bit '

c. Yes, discreetly

d. Yes, very

17) Would you take part in public meetings held by other patients sharing their experiences?

a. Yes, only with people with the same problem

b. Yes, but with the presence of the neurologist

c. Yes, but with the presence of the psychologist

d. No
